# Genome Sequence of Growth-Improving *Paenibacillus mucilaginosus* Strain KNP414

**DOI:** 10.1128/genomeA.00881-13

**Published:** 2013-10-24

**Authors:** Jing-Jiang Lu, Jian-Feng Wang, Xiu-Fang Hu

**Affiliations:** College of Life Science, Zhejiang Sci-Tech University, Xiasha, Hangzhou, People’s Republic of China

## Abstract

*Paenibacillus mucilaginosus* is a critical growth-improving silicate bacterium. Here, we report the complete genome sequence of *P. mucilaginosus* strain KNP414. This information will provide us with the opportunity to understand its molecular mechanisms and develop more effective utilization of the strain.

## GENOME ANNOUNCEMENT

*Paenibacillus mucilaginosus* is a silicate bacterium (1) that is Gram positive, facultatively anaerobic, and spore forming (2) and is widely distributed in the soil, rhizosphere, and other locations (3). *P. mucilaginosus* strain KNP414 forms a large number of capsular polysaccharides when cultured in nitrogen-free medium and produces organic acid (4). It is able to degrade insoluble soil minerals (4), release nutritional ions (5), and fix nitrogen (6). Because of its many functions, *P. mucilaginosus* KNP414 is widely used in agriculture as a biofertilizer. Here, we report the complete genome sequence of *P. mucilaginosus* KNP414. To date, only two genome sequences, those of *P. mucilaginosus* strains K02 (GenBank accession no. NC_017672) and 3016 (accession no. NC_016935), have been completed for *P. mucilaginosus*.

The genomic DNA was isolated from cells of *P. mucilaginosus* KNP414 after culturing in Aleksandrov medium with 0.2% (NH_4_)_2_SO_4_ using a DNA isolation kit (catalog no. 17900, bacterial genomic DNA isolation kit; Norgen, Canada). We constructed three genome libraries (400 bp, 2 to ~3 kb, and 4 to ~9 kb) and sequenced using the Illumina Solexa genome analyzer II platform. In total, 15,510,396 reads were obtained with 126-fold sequencing depth, and the accuracy rate of the sequences was 99.82%. Illumina reads were assembled using Velvet (7), ABySS (8) and SOAP*denovo* (9), and the scaffolds were contrasted using SOAP*denovo*. The genome was finished by amplifying across gaps using information of the super genome library (4 to ~9 kb) and PCR.

The *P. mucilaginosus* KNP414 genome consists of a circular chromosome (8,663,821 bp) with a 58.38% G+C content, which is similar to the other two sequenced *P. mucilaginosus* strains (K02 and 3016), and their genomic G+C contents are a little higher than those of most *Paenibacillus* strains (45 to 54%) in accordance with a previous report (10). The replication origin was predicted at position 8663004 using Ori-Finder (11) (http://tubic.tju.edu.cn/Ori-Finder/), and the potential coding sequences (CDSs) were predicted using GeneMark and Glimmer. The KNP414 genome encodes 7,811 potential proteins, with 85% coding density. Among these genes, 4,608 (58.99%) are assigned to encode known proteins, whereas 3,203 (41.01%) are identified to encode hypothetical proteins, which do not have BLASTp matches to any protein entries in the NR or UniProt database with an *E* value cutoff of e^-10^. In addition, the genome contains 13 rRNA operons, 107 tRNAs, and 6 potential small RNAs (sRNAs) based on the conserved sequence features in the intergenic regions identified using RfamScan (12); all of these values are lower than those for the other two *P. mucilaginosus* strains.

The genome of *P. mucilaginosus* KNP414 harbors eight genes related to nitrogen assimilation, and a comparison analysis shows that most of these genes are specifically distributed in the genome. These genes make KNP414 able to fix nitrogen and grow in a nitrogen-free environment. However, the mechanisms for this are unknown. Further analysis of the KNP414 genome will advance our understanding of the molecular mechanisms and develop more effective utilization of the strain.

### Nucleotide sequence accession number.

The sequence of *P. mucilaginosus* KNP414 has been deposited at GenBank under the accession no. CP002869.
